# Comparison of sarcopenia prevalence and prognostic features between HFrEF and HFpEF: a systematic review and meta-analysis

**DOI:** 10.3389/fcvm.2025.1671305

**Published:** 2025-11-17

**Authors:** Weizhu Xiong, Jun Yang, Xinbin Zhou, Siyin Wang, Jin Dai, Xiao Wang

**Affiliations:** 1 Department of Cardiology, The First Affiliated Hospital of Zhejiang Chinese Medical University (Zhejiang Provincial Hospital of Chinese Medicine), Hangzhou, Zhejiang, China; 2 Department of Geriatrics, The First Affiliated Hospital of Zhejiang Chinese Medical University (Zhejiang Provincial Hospital of Chinese Medicine), Hangzhou, Zhejiang, China

**Keywords:** heart failure, sarcopenia, ejection fraction, meta - analysis, systematic review

## Abstract

**Background:**

Sarcopenia is closely associated with heart failure (HF); however, no prior meta-analysis has specifically addressed its relation with different ejection fraction phenotypes. This study investigated the prevalence of sarcopenia in patients with HF with reduced ejection fraction (HFrEF) vs. those with preserved ejection fraction (HFpEF), compared their prevalence rates, and explored the prognostic outcomes associated with sarcopenia in these phenotypes.

**Methods:**

PubMed, Cochrane, and Embase databases were searched from their inception to February 2025. Studies reporting the prevalence or prognosis of sarcopenia in patients with HF and defined ejection fraction phenotypes were included. Two authors independently assessed study quality using the Newcastle–Ottawa Scale and Agency for Healthcare Research and Quality. Meta-analyses were conducted using Stata 17, with random-effects models applied to heterogeneous data.

**Results:**

Twenty studies were included: 17 on sarcopenia prevalence in HFrEF, four in HFpEF, four comparing the prevalence between phenotypes, and two comparing prognoses. The pooled prevalence rate of sarcopenia was 35% and 28% in patients with HFrEF and HFpEF, respectively. Subgroup analyses revealed regional variations: Asian populations showed a higher prevalence in HFrEF (48%) that that in HFpEF (16%), whereas European populations exhibited a higher prevalence in HFpEF (44%) than that in HFrEF (27%). In America, the prevalence of sarcopenia in patients with HFrEF was 29%. Age-stratified analyses demonstrated a sarcopenia prevalence of 30% in patients with HFrEF aged ≥65 years vs. 36% in those <65 years. Hospitalized patients with HFrEF had a higher prevalence (45%) than that of the outpatient cohort (23%), whereas hospitalized patients with HFpEF showed a 43% prevalence vs. 16% in outpatients. A meta-analysis of studies directly comparing HFrEF and HFpEF found no significant difference in sarcopenia prevalence (fixed-effect model: RR = 1.12, 95% CI: 1.01–1.23; *I*^2^ = 23%, *p* = 0.273). Prognostic comparisons between patients with sarcopenic HFrEF and HFpEF also showed no significant difference (hazard ratio = 1.57, 95% CI: 0.66–3.77; *I*^2^ = 79%, *p* = 0.029).

**Conclusion:**

In epidemiology, the prevalence of sarcopenia was higher in patients with HFrEF than in those with HFpEF. However, Among studies that include a comparison of the prevalence rates of HFrEF and HFpEF with sarcopenia, meta-analyses have indicated that the ejection fraction phenotype is neither associated with the prevalence of sarcopenia in HF nor with poor outcomes in patients with HF and sarcopenia.

**Systematic Review Registration:**

https://www.crd.york.ac.uk/PROSPERO/view/CRD420251077599, PROSPERO CRD420251077599.

## 1 Introduction

Heart failure (HF) is a chronic syndrome characterized by dyspnea and fatigue (1). According to the 2025 report by the World Health Organization (WHO), the total number of global heart failure (HF) patients has exceeded 64 million, and the prevalence rate is still increasing at a rate of approximately 2% per year. Sarcopenia, an age-related loss of skeletal muscle mass and function, the prevalence of sarcopenia among people aged 60 years and above worldwide is 10%–27%, exhibits a prevalence and progression strongly linked to comorbid risk factors. The development of sarcopenia in patients with heart failure (HF) is associated with multiple mechanisms (2). Although recent clinical trials and reviews have evaluated the clinical outcomes and prevalence of sarcopenia in HF populations (3, 4), epidemiological studies specifically comparing sarcopenia across distinct ejection fraction (EF) phenotypes—reduced (HFrEF) and preserved (HFpEF)—remain scarce, limiting accurate prevalence estimation. Elucidating the prevalence of sarcopenia across these phenotypes is a critical research priority.

A study systematically assessed the prevalence of sarcopenia in patients with HFrEF and HFpEF (3). However, its limited sample size precluded robust conclusions, reporting pooled prevalences of 28% (95% CI: 0.17–0.38; I^2^ = 96%, *p* < 0.01) for HFrEF and 18% (95% CI: 0.15–0.22; I^2^ = 0.0%, *p* < 0.01) for HFpEF. Furthermore, no meta-analysis has directly compared the prevalence of sarcopenia between these phenotypes or evaluated the prognosis of patients with sarcopenic HFrEF vs. those with sarcopenic HFpEF.

Given these inconsistencies and research gaps, we conducted this meta-analysis to (1) characterize sarcopenia epidemiology across HF phenotypes, (2) compare the prevalence of sarcopenia in patients with HFrEF and HFpEF, and (3) assess the prognostic impact of EF phenotype in patients with sarcopenic HF.

## 2 Methods

The protocol of this network meta-analysis was registered in the PROSPERO (CRD420251077599).

### 2.1 Search strategy

We systematically searched PubMed, Cochrane Library, and Embase from inception to February 2025 using MeSH terms: “*Heart Failure*”, “*Cardiac Failure*”, “*Myocardial Failure*”, “*ejection fraction*”, and “*Sarcopenia*”. Two investigators (WZX and XBZ) independently executed the search and cross-verified results to ensure accuracy.

### 2.2 Study selection

Retrieved articles underwent title/abstract screening adhering to the “independent parallel review” principle. Two investigators (WZX and XBZ) independently applied predefined eligibility criteria. The full texts of potentially relevant studies were reviewed for final inclusion. Discrepancies were resolved through discussions with a third investigator (Jin Dai) until consensus was reached.

### 2.3 Inclusion and exclusion criteria

The inclusion criteria were as follows: (1) original studies (cross-sectional or cohort design) reporting the clinical outcomes of patients with sarcopenia and HF of any sex or ethnicity, (2) clear differentiation between HFrEF and HFpEF, (3) explicit sarcopenia diagnosis using validated criteria, and (4) reporting phenotype-specific sarcopenia prevalence and clinical outcomes. The exclusion criteria included studies that failed to stratify HF by EF, non-original data (reviews, letters, conference abstracts, and case reports), animal studies, or non-English publications.

### 2.4 Data extraction

Two investigators (WZX and XBZ) independently extracted data, including publication year, study design, population demographics (age and sex), sample size, HF phenotype, sarcopenia diagnostic criteria, prevalence, and clinical outcomes, using standardized forms. The extracted data were cross-checked, and discrepancies were resolved via consensus.

### 2.5 Risk of bias assessment

Cohort studies were evaluated using the Newcastle–Ottawa Scale (NOS), which assesses selection, comparability, and outcome domains (maximum nine stars; ≥7 indicating high quality). Cross-sectional studies were appraised via the 11-item checklist from the Agency for Healthcare Research and Quality (AHRQ), with scores ≥7/11 denoting high quality. Two reviewers (WZX and XBZ) independently conducted the assessments and resolved any disagreements through discussion.

### 2.6 Statistical analysis

Data analysis was performed using Stata 17.0 software. Heterogeneity across studies was assessed via Cochran's Q statistic and the *I*^2^ index, with *I*^2^ ≥ 50% indicating substantial heterogeneity. A *p*-value ≤0.05 was considered statistically significant. For the pooled analyses, random-effects models were applied when significant heterogeneity was detected to generate conservative estimates; otherwise, fixed-effects models were used. Forest plots illustrated pooled results. To explore the sources of heterogeneity in sarcopenia prevalence across EF phenotypes, subgroup and meta-regression analyses were conducted based on region, age, population source (including hospitalized vs. outpatient), and sarcopenia diagnostic criteria. Publication bias was evaluated using Begg's rank correlation test and Egger's regression test. Sensitivity analyses were performed to assess the robustness of the pooled estimates.

## 3 Results

### 3.1 Study selection

In total, 2,606 articles were identified through systematic searches of PubMed, Embase, and the Cochrane Library, supplemented by seven additional records from prior meta-analyses on HF and sarcopenia. After removing 644 duplicates, the titles/abstracts of 1,962 articles were screened, yielding 75 full-text reviews. Of these, 55 were excluded because of irrelevance (including non-English publications, review articles, conference abstracts, letters, or insufficient data). Finally, 20 studies that met the inclusion criteria were included. A detailed flowchart of the literature retrieval and selection processes is shown in Supplementary Figure S1.

### 3.2 Characteristics of the included studies

Table 1 summarizes the detailed characteristics of the 20 included studies, comprising 8 cross-sectional and 12 cohort studies. These studies, published from database inception to February 2025, enrolled 5,031 patients with HF and well-characterized EF phenotypes. Among them, 17 studies reported the prevalence of sarcopenia in patients with HFrEF, four studies focused on sarcopenia prevalence in patients with HFpEF, four studies concurrently assessed both HFrEF and HFpEF populations, and one study lacked prevalence data but compared prognostic outcomes between patients with HFrEF and sarcopenia and those with HFpEF and sarcopenia. Geographically, nine studies involved Asian populations, five were conducted in the Americas, and six were conducted in Europe. The study populations consisted of hospitalized patients (10 studies) and outpatients (10 studies). Sarcopenia was diagnosed using the Asian Working Group for Sarcopenia criteria in nine studies and the European Working Group on Sarcopenia in Older People criteria in six studies.

**Table 1:** Characteristics of the included studies.

Authors (year)	Region	Study design	Sample number	Meanage (SD)	Participants	Type of HF	Definition of sarcopenia	Rate%	SE
Masaaki Konishi (2020) A (5)	Japan	Multicentre prospective cohort study	475	81 (7)	Hospitalized patients	HFpEF	AWGS	0.181	0.017
Masaaki Konishi (2020) B (5)	Japan	Multicentre prospective cohort study	467	78 (8)	Hospitalized patients	HFrEF	AWGS	0.216	0.019
Yasutaka Imamura (2024) (6)	Japan	Retrospective cohort study	256	85 (9)	Hospitalized patients	AHF	AWGS		
Masaaki Konishi (2021) A (7)	Japan	Observational cohort study	193	85 (11)	Hospitalized patients	HFpEF	AWGS	0.482	0.036
Masaaki Konishi (2021) B (7)	Japan	Observational cohort study	225	68 (14)	Hospitalized patients	HFrEF	AWGS	0.582	0.033
Tamirys (2023) (8)	Brazil	Cross-sectional study	90	69.4 (7.2)	Outpatients	HFrEF	EWGSOP2	0.244	0.045
Satoshi Katano (2022) (9)	Japan	Retrospective cohort study	539	72 (14)	Hospitalized patients	HFrEF (HFpEF)	AWGS	0.651 (0.606)	0.035 (0.026)
Hayley E Billingsley (2022) (10)	America	Cross-sectional study	40	57 (10)	Outpatients	HFrEF	EWGSOP	0.425	0.078
RuiXu (2022) (11)	China	Cross-sectional observational study	80	76 (1.8)	Hospitalized patients	HFrEF	AWGS	0.5	0.056
Yousuke Sugita (2023) (12)	Japan	Cross-sectional study	99	74 (5)	Outpatients	HFpEF	AWGS	0.121	0.033
D. Fonseca (2020) (13)	Brazil	Cross-sectional study	168	57 (8.2)	Outpatients	HFrEF	Others	0.393	0.038
Andre L Canteri (2019) (14)	Brazil	Cross-sectional study	79	65.6 (13)	Outpatients	HFrEF	EWGSOP	0.101	0.034
Marcelo R Dos Santos (2017) (15)	Germany	Cross-sectional study	228	68.8 (9.6)	Outpatients	HFrEF	EWGSOP	0.195	0.026
Masakazu Saitoh (2016) (16)	Germany	Retrospective cohort study	130	66.3 (11.5)	Outpatients	HFrEF	Others	0.146	0.031
Amir Emami (2018) (17)	Germany	Prospective cohort study	207	67.3 (10.1)	Outpatients	HFrEF	AWGS	0.213	0.028
Tarek Bekfani (2016) (18)	Germany	Cross-sectional study	117	69.8 (8.5)	Outpatients	HFpEF	Others	0.197	0.037
Wenxue Zhao (2020) (19)	China	Cross-sectional study	355	71 (9.4)	Hospitalized patients	HFrEF	AWGS	0.558	0.026
DaFonseca (2019) (20)	Germany	Cross-sectional study	116	55 (9)	Hospitalized patients	HFrEF	European Working Group	0.284	0.042
Romain Eschalier (2020) (21)	France	Prospective cohort study	140	75.8 (10.2)	Hospitalized patients	HFrEF (HFpEF)	EWGSOP	00.626 (0.687)	00.051 (0.067)
Persio D. Lopez (2019) (22)	USA	Retrospective cohort study	160	66.3 (13.8)	Hospitalized patients	HFrEF	Others	0.325	0.037
Satoshi Katano (2024) (23)	Japan	Ambispective cohort study	145		Hospitalized patients	HFrEF	AWGS	0.359	0.039
Raif Kılıc (2024) (24)	Turkey	Retrospective cohort study	722	70.1 (8.4)	Outpatients	HFrEF	Others	0.234	0.016

HF, heart failure; HFpEF, heart failure with preserved ejection fraction; HFrEF, heart failure with reduced ejection fraction; AWGS, Asian Working Group for Sarcopenia; EWGSOP, European Working Group on Sarcopenia in Older People.

### 3.3 Risk of bias in included studies

Details to be added based on NOS and AHRQ assessments, including “The majority of cohort studies (*n* = 11) scored ≥7 on the NOS, indicating low risk of bias. Cross-sectional studies (*n* = 3) achieved AHRQ scores ≥7/11, suggesting high methodological quality”, as shown in Supplementary Tables S1, S2.

### 3.4 Epidemiology of sarcopenia in patients with HFrEF and HFpEF

**Figure 1:**
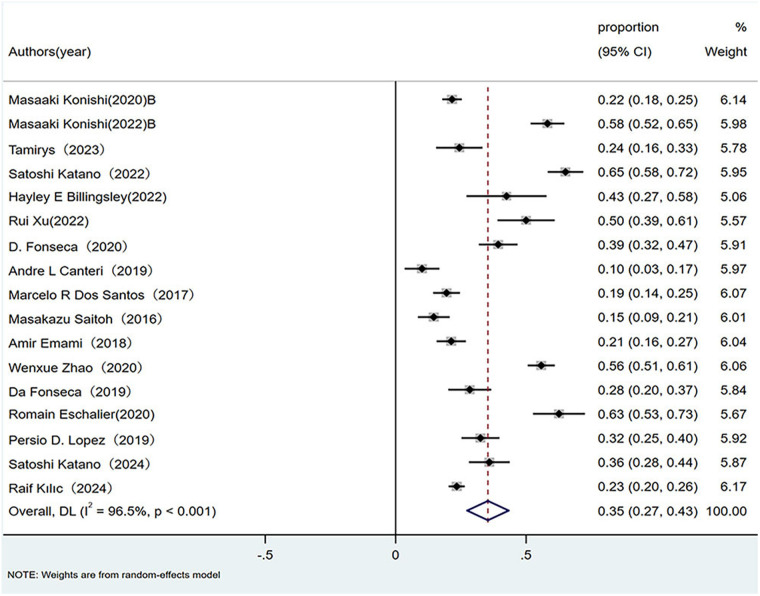
Prevalence of sarcopenia in patients with HFrEF. HFrEF, Heart failure with reduced ejection fraction.

Twenty observational studies evaluated the epidemiology of sarcopenia across HF phenotypes. Pooled prevalence was calculated using random-effects models because of significant heterogeneity. As illustrated in the forest plot, the overall sarcopenia prevalence in patients with HFrEF was 35% (95% CI: 0.27–0.43; *p* < 0.01), with substantial heterogeneity (*I*^2^ = 96.5%, *p* < 0.01), as shown in Figure 1.

**Figure 2:**
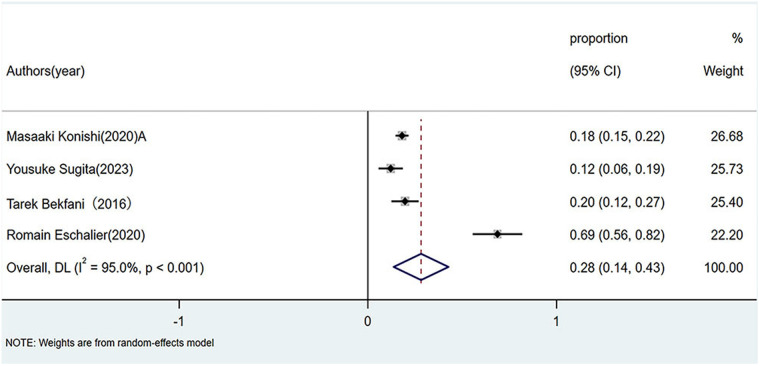
Prevalence of sarcopenia in patients with HFpEF. HFpEF, Heart failure with preserved ejection fraction.

Patients with HFpEF exhibited a pooled sarcopenia prevalence of 28% (95% CI: 0.14–0.43; *p* < 0.01), also marked by high heterogeneity (*I*^2^ = 95%, *p* < 0.01), as shown in Figure 2.

### 3.5 Subgroup analysis and meta-regression

Given the substantial heterogeneity in the pooled estimates, subgroup and meta-regression analyses were conducted to explore the potential sources of variability in the prevalence of sarcopenia across HF phenotypes. Stratifications were based on region, age, diagnostic criteria for sarcopenia, and population source (hospitalized vs. outpatient).

Regional stratification revealed the following: Asian populations: HFrEF sarcopenia prevalence = 48% (95% CI: 0.31–0.64; *I*^2^ = 97.7%, *p* < 0.01); HFpEF prevalence = 16% (95% CI: 0.10–0.21; *I*^2^ = 61.5%, *p* < 0.01).European populations: HFrEF prevalence = 27% (95% CI: 0.19–0.36; *I*^2^ = 93.1%, *p* < 0.01); HFpEF prevalence = 44% (95% CI: −0.04–0.92; *I*^2^ = 93.1%, *p* < 0.01). American populations: HFrEF prevalence = 29% (95% CI: 0.17–0.41; *I*^2^ = 90.5%, *p* < 0.01).
Age-stratified analysis demonstrated: HFrEF patients ≥65 years: Prevalence = 30% (95% CI: 0.21–0.39; *I*^2^ = 96.6%, *p* < 0.01). HFrEF patients <65 years: Prevalence = 36% (95% CI: 0.27–0.44; *I*^2^ = 57.3%, *p* < 0.01).
Diagnostic criteria stratification: AWGS criteria: HFrEF prevalence = 44% (95% CI: 0.29–0.59; *I*^2^ = 97.7%, *p* < 0.01).EWGSOP criteria: HFrEF prevalence = 31% (95% CI: 0.17–0.44; *I*^2^ = 94.1%, *p* < 0.01).Other criteria: HFrEF prevalence = 27% (95% CI: 0.18–0.36; *I*^2^ = 90.3%, *p* < 0.01).
Population source stratification: Hospitalized patients: HFrEF prevalence = 45% (95% CI: 0.33–0.58; *I*^2^ = 96.8%, *p* < 0.01); HFpEF prevalence = 43% (95% CI: −0.07–0.93; *I*^2^ = 98.1%, *p* < 0.01). Outpatients: HFrEFprevalence = 23% (95% CI: 0.17–0.29; *I*^2^ = 85.4%, *p* < 0.01); HFpEF prevalence = 16% (95%CI:0.08–0.23; *I*^2^ = 57.9%, *p* < 0.01). (see Supplementary Table S3).

Meta-regression identified population source as a significant contributor to heterogeneity in patients with HFrEF (*p* = 0.045) (Tables 2, 3). No other variables significantly explained the heterogeneity across subgroups.

**Table 2:** Univariable meta-regression analysis of sarcopenia prevalence in HFrEF patients.

Variable	Coefficient	SE	*t*	*P* > ltl	95% CI
Region	−0.16	0.05	−0.32	0.754	−0.13–0.99
Age	−0.09	0.1	−0.94	0.376	−0.32–0.13
Definition of sarcopenia	−0.03	0.05	−0.72	0.49	−0.15–0.08
Participants	−0.18	0.07	−2.37	0.04	−0.36 to −0.00
-Cons	0.77	0.26	2.89	0.02	0.15–1.38

**Table 3:** Univariable meta-regression analysis of sarcopenia prevalence in HFpEF patients.

Variable	Coefficient	SE	*t*	*P* > ltl	95% CI
Region	0.28	0.21	1.34	0.4	−2.44–3.01
Participants	−0.26	0.21	−1.26	0.42	−3.00–2.46
-Cons	0.266	0.46	0.58	0.66	−5.61–6.14

### 3.6 Publication bias assessment

Begg's rank correlation test and Egger's regression test revealed no significant publication bias in the meta-analysis of sarcopenia prevalence among patients with HFrEF and HFpEF (*p* > 0.10 for both tests), indicating stable pooled estimates (as illustrated in Supplementary Figure S3).

### 3.7 Sensitivity analysis

Sensitivity analyses were conducted by sequentially excluding individual studies and recalculating pooled prevalence estimates. The results demonstrated no substantial alterations in the effect sizes for either the HFrEF or HFpEF cohorts, confirming the robustness of the findings (Supporting Information: Supplementary Figure S2).

### 3.8 Comparison of sarcopenia prevalence between HFrEF and HFpEF

Four studies that directly compared the prevalence of sarcopenia between the HFrEF and HFpEF groups were analyzed using both random- and fixed-effects models. Because of missing data on sarcopenia prevalence in patients with HFpEF [left ventricular EF (LVEF) ≥50%] in two studies, subgroup analyses were performed under two definitions: (1) HFrEF as LVEF <50% (5, 21) and (2) HFrEF as LVEF <40% (7, 9).

Random-effects model: LVEF <50%: RR 1.04 (95% CI: 0.80–1.36; *I*^2^ = 54.6%, *p* = 0.138). LVEF <40%: RR 1.12 (95% CI: 1.00–1.25; *I*^2^ = 2.4%, *p* = 0.312). Fixed-effect model: LVEF <50%: RR 1.10 (95% CI: 0.91–1.33; *I*^2^ = 61.5%, *p* = 0.107). LVEF <40%: RR 1.13 (95% CI: 1.01–1.26; *I*^2^ = 4.3%, *p* = 0.307).

**Figure 3:**
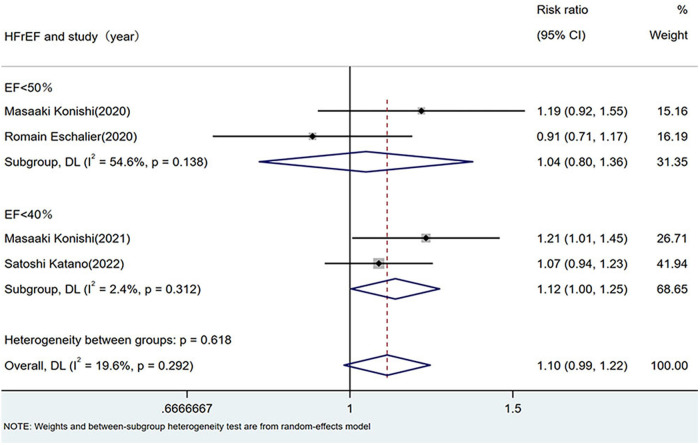
Comparison of sarcopenia prevalence between HFrEF and HFpEF. HF, heart failure; HFpEF, heart failure with preserved ejection fraction; HFrEF, heart failure with reduced ejection fraction.

Both definitions yielded non-significant differences in sarcopenia prevalence between the HFrEF and HFpEF groups, as shown in Figure 3.

### 3.9 Impact of EF phenotype on prognosis in patients with HF and sarcopenia

**Figure 4:**
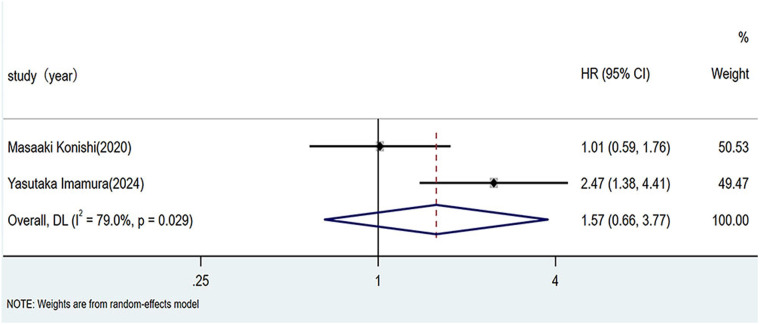
Impact of ejection fraction phenotype on prognosis in HF patients with sarcopenia. HF, heart failure.

As illustrated in Figure 4, pooled analysis using a random-effects model revealed a hazard ratio of 1.57 (95% CI: 0.66–3.77; *I*^2^ = 79%, *p* = 0.029) for adverse prognosis in patients with HF and sarcopenia across EF phenotypes. The non-significant association (*p* > 0.01) indicated that the EF phenotype (HFrEF vs. HFpEF) did not significantly influence the risk of poor prognosis in patients with sarcopenic HF.

## 4 Discussion

In this study, we systematically investigated the epidemiology of sarcopenia prevalence among patients with HF stratified by EF phenotype (HFrEF/HFpEF), performed subgroup analyses, compared interphenotype prevalence rates, and assessed the prognostic implications of EF phenotypes in patients with HF and comorbid sarcopenia. By synthesizing extensive research data through rigorous meta-analytic methods (random-effects model with inverse-variance weighting), we identified the following key outcomes: Epidemiological analysis revealed sarcopenia prevalence rates of 0.35 (95% CI: 0.28–0.42) in patients with HFrEF and 0.28 (95% CI: 0.21–0.35) in those with HFpEF. However, most of the studies included in the analysis only reported the prevalence of sarcopenia in HFrEF or only in HFpEF, The between-group heterogeneity in such comparisons is excessively high, making the results not robust. We have conducted further research and found that no significant difference among studies that include a comparison of the prevalence of sarcopenia in both HFrEF and HFpEF was observed. Additionally, the EF phenotype was not independently associated with an increased risk of adverse prognosis in patients with HF and sarcopenia. Evidence-based clinical guidance: Universal sarcopenia prevention protocols should be implemented in all patients with HF, irrespective of EF phenotype (HFrEF vs. HFpEF), to attenuate sarcopenia-associated prognostic deterioration, thereby enhancing survival rates and health-related quality of life. Aggressive sarcopenia management is imperative in patients with HF diagnosed with sarcopenia across all EF subgroups, as prognosis remains unaffected by parameters of left ventricular systolic function.

Prior meta-analyses have reported sarcopenia prevalence rates of 0.28 in patients with HFrEF and 0.18 in those with HFpEF (3). However, these estimates are limited by inadequate sample sizes and methodological constraints. In this updated analysis with expanded cohort enrollment, we conducted rigorous epidemiological re-evaluations, revealing significantly elevated sarcopenia prevalence rates of 0.35 (95% CI: 0.30–0.40) in HFrEF and 0.28 (95% CI: 0.23–0.33) in HFpEF. Notably, both phenotypes demonstrated an increased sarcopenia burden compared to historical data. The prevalence of sarcopenia was consistently higher in patients with HFrEF than in those with HFpEF in both analyses. The higher sarcopenia prevalence in patients with HFrEF than in those with HFpEF is hypothesized to be attributable to the following factors: HFrEF leads to a more severe reduction in peripheral blood flow than HFpEF, which more significantly limits patients' exercise capacity, thereby directly or indirectly causing greater loss of muscle mass than is observed in patients with HFpEF. Additionally, the chronic increase in vascular resistance induced by HF results in reduced skeletal muscle perfusion and hypoxia, leading to the accumulation of metabolic by-products that activate metabolic reflexes and trigger the development of sarcopenia (25).

Subgroup analyses of sarcopenia prevalence in patients with HFrEF and HFpEF have not been conducted in previous studies (3). In this study, subgroup analyses were performed for horizontal and vertical comparisons. Stratified analysis by region showed that the prevalence of sarcopenia in patients with HFrEF was lower than that in those with HFpEF among European populations. We hypothesize that this may be because of insufficient inclusion of studies and substantial heterogeneity in the European HFpEF sarcopenia subgroup, or because European countries provide higher-level healthcare measures with greater emphasis on early interventions for patients with HFrEF, thereby reducing the incidence of sarcopenia. The prevalence of sarcopenia in patients with HFrEF was higher in Asian populations than in European and American populations. This may be due to the inclusion of primarily developing nations in the Asian subgroup, which tend to have lower nutritional levels than those of developed countries, resulting in muscle damage that affects both muscle mass and function (26). Alternatively, it may be attributable to more advanced healthcare systems and higher awareness of sarcopenia in developed countries, enabling earlier interventions to reduce sarcopenia prevalence, whereas hospitals in many developing countries still face significant limitations in recognizing and managing sarcopenia.

Subgroup analysis by different age groups showed that the prevalence of sarcopenia in patients with HFrEF aged ≥65 years was lower than that in those aged <65 years. This finding differs from those of previous studies (3, 27), and suggests that age may play a significant role in the risk of sarcopenia. Although prior research has indicated that the prevalence of sarcopenia increases with age and that the decline in skeletal muscle mass, strength, and function with aging appears indisputable, the opposite was observed in patients with HFrEF. This may be because of faster disease progression and greater muscle damage in younger patients with HFrEF, or may be related to the smaller sample size of younger participants in this subgroup (10). The exact cause of this discrepancy remains unclear and requires further clinical investigation. This highlights the need for clinicians to prioritize sarcopenia screening and early intervention in younger patients with HFrEF to mitigate disease progression.

Subgroup analysis using different sarcopenia diagnostic criteria revealed that the prevalence of sarcopenia diagnosed by Asian Working Group for Sarcopenia in patients with HFrEF was higher than that diagnosed by European Working Group on Sarcopenia in Older People or other criteria. This finding aligns with the results of the regional stratified analyses and is possibly attributable to the geographic specificity of different diagnostic standards (3).

Subgroup analysis by population source (inpatient vs. outpatient) showed a similar prevalence of sarcopenia between inpatients with HFrEF and those with HFpEF, whereas patients with HFrEF had a higher prevalence than those with HFpEF among outpatients. This may be because inpatients, who generally have more severe conditions, commonly exhibit sarcopenia, thereby blurring the difference in prevalence between the HFrEF and HFpEF groups. Additionally, the prevalence of sarcopenia was higher in inpatients than in outpatients for both HFrEF and HFpEF, likely because of milder symptoms and lower severity of HF-related sarcopenia during ambulatory diagnosis and treatment. These findings represent the results of the epidemiological subgroup analyses of sarcopenia prevalence in the HFrEF and HFpEF groups.

Lower EF in patients is associated with a higher prevalence of sarcopenia (3). In the current analysis, we compared the prevalence of sarcopenia between patients with HFrEF and those with HFpEF using data from studies reporting both phenotypes. A meta-analysis revealed no significant difference in the prevalence of sarcopenia between the two groups, indicating that EF phenotype has no impact on the prevalence of sarcopenia in patients with HF (5, 7, 9, 21). This finding diverges from prior research, and the discrepancy may be attributable to the possibility that reduced EF is not the primary factor responsible for increased sarcopenia prevalence in this population.

Additionally, we investigated the relation between EF phenotypes and the prognosis of patients with HF and sarcopenia. While some studies have suggested that reduced EF is associated with an increased risk of adverse outcomes in this population (6), others have contradicted this view (5). In the present study, no significant association was found between EF phenotype and adverse outcomes in patients with HF and sarcopenia. However, there is a paucity of studies directly comparing the prognoses of patients with HFrEF and HFpEF with sarcopenia. Regarding outcome measures comparing the prognosis of patients with HF with different EF phenotypes and comorbid sarcopenia, only two studies met the inclusion criteria. The pooled analysis did not reveal any statistically significant differences. Nonetheless, these findings should be interpreted with caution. First, the number of included studies was small, and significant heterogeneity was observed. Second, the lack of significant differences may be attributable to short follow-up durations and/or the inclusion of patients with HFrEF defined using an EF threshold of <45%. Therefore, the current evidence is insufficient to conclude that there is no prognostic difference between these groups. Further largescale research is needed to elucidate the relation between EF and prognosis in patients with HF and sarcopenia. Such investigations will guide clinicians in implementing timely interventions to prevent sarcopenia in patients with HF across different EF phenotypes, thereby improving prognosis and quality of life, while alleviating the burden on families and society.

### 4.1 Strengths and limitations

This study had several strengths and limitations. Its primary strength lies in the systematic meta-analysis of sarcopenia prevalence in patients with HFrEF and HFpEF. We addressed a critical gap by expanding the sample size and refining statistical methods, thereby enabling both horizontal and vertical epidemiological comparisons of sarcopenia prevalence across different HF phenotypes. Additionally, we conducted a novel meta-analysis comparing sarcopenia prevalence in patients with HFrEF and those with HFpEF using studies that reported both phenotypes, thus filling an important knowledge gap. Furthermore, although previous research suggested a worse prognosis in patients with HFrEF and sarcopenia compared to those with HFpEF, our meta-analysis—the first of its kind—revealed no significant difference in outcomes between the two phenotypes. We employed univariate regression analysis to explore heterogeneity and enhance the robustness of our findings.

However, this study had several limitations. First, all included studies were observational in design and subject to potential bias due to subjective assessment. Second, the exclusive inclusion of English-language publications may have resulted in underrepresentation of data reported in other languages. Finally, In the epidemiological analysis of this study, the overall heterogeneity tests for the prevalence of sarcopenia in HFrEF and HFpEF separately showed I^2^ = 96.5% and I^2^ = 95%, indicating significant heterogeneity. First, we conducted subgroup analyses stratified by region, age, population source, and sarcopenia diagnostic criteria; the results showed that heterogeneity remained high within each subgroup. We further analyzed the potential reasons for the failure to reduce heterogeneity after subgroup analysis: (1). Some potential heterogeneity dimensions have not been fully covered, which may lead to unidentified baseline differences remaining among the population within subgroups; (2). There is an overlap of multiple heterogeneities (e.g., the interactive effect of “region + population activity level”), making it difficult to completely disentangle them through single-dimensional subgroup analyses; (3). A small number of small-sample studies may have increased the heterogeneity fluctuation within subgroups. Given that there is currently a limited number of existing studies and some data collection is difficult, future studies should further expand the sample size and refine the baseline population characteristics; furthermore, we will continue to follow up on this issue in the future. The relative paucity of studies comparing sarcopenia prevalence and prognosis across HF phenotypes warrants caution in interpreting the results of this meta-analysis, necessitating further validation through additional clinical research. Future studies should also investigate the complex mechanisms underlying the association between HF phenotypes and sarcopenia to identify targeted, phenotype-specific interventions.

## 5 Conclusion

Epidemiologically, sarcopenia prevalence is higher in patients with HFrEF than in those with HFpEF. However, among studies that include a comparison of the prevalence of sarcopenia in both HFrEF and HFpEF indicate that EF phenotype is neither associated with sarcopenia prevalence in HF nor with adverse outcomes in patients with HF and sarcopenia.

## Data Availability

The datasets presented in this study can be found in online repositories. The names of the repository/repositories and accession number(s) can be found in the article/Supplementary Material.

## References

[1] Mosterd A Hoes AW. Clinical epidemiology of heart failure. Heart. (2007) 93:1137–46. 10.1136/hrt.2003.025270 17699180 PMC1955040

[2] Doehner W Turhan G Leyva F Rauchhaus M Sandek A Jankowska EA Skeletal muscle weakness is related to insulin resistance in patients with chronic heart failure. ESC Heart Fail. (2015) 2:85–9. 10.1002/ehf2.12035 28834658 PMC6410535

[3] Chen R Xu J Wang Y Jiang B Xu X Lan Y Prevalence of sarcopenia and its association with clinical outcomes in heart failure: an updated meta-analysis and systematic review. Clin Cardiol. (2023) 46:260–8. 10.1002/clc.23970 36644878 PMC10018088

[4] Liu Y Su M Lei Y Tian J Zhang L Xu D. Sarcopenia predicts adverse prognosis in patients with heart failure: a systematic review and meta-analysis. Rev Cardiovasc Med. (2023) 24:273. 10.31083/j.rcm2409273 39076387 PMC11270102

[5] Konishi M Kagiyama N Kamiya K Saito H Saito K Ogasahara Y Impact of sarcopenia on prognosis in patients with heart failure with reduced and preserved ejection fraction. Eur J Prev Cardiol. (2021) 28:1022–9. 10.1093/eurjpc/zwaa117 33624112

[6] Imamura Y Suzuki A Kamishima K Suzuki K Yamaguchi J. Prognostic factors in patients with heart failure and sarcopenia: an observational retrospective study. Egypt Heart J. (2024) 76:52. 10.1186/s43044-024-00484-4 38683441 PMC11058133

[7] Konishi M Akiyama E Matsuzawa Y Sato R Kikuchi S Nakahashi H Prognostic impact of muscle and fat mass in patients with heart failure. J Cachexia Sarcopenia Muscle. (2021) 12:568–76. 10.1002/jcsm.12702 33939328 PMC8200420

[8] Sangali TD Souza GC Ribeiro ÉCT Perry IDS. Sarcopenia: inflammatory and humoral markers in older heart failure patients. Arq Bras Cardiol. (2023) 120:e20220369. 10.36660/abc.20220369 37556651 PMC10382140

[9] Katano S Honma S Nagaoka R Numazawa R Yamano K Fujisawa Y Anthropometric parameters-derived estimation of muscle mass predicts all-cause mortality in heart failure patients. ESC Heart Fail. (2022) 9:4358–65. 10.1002/ehf2.14121 36065759 PMC9773643

[10] Billingsley HE Del Buono MG Canada JM Kim Y Damonte JI Trankle CR Sarcopenic obesity is associated with reduced cardiorespiratory fitness compared with nonsarcopenic obesity in patients with heart failure with reduced ejection fraction. Circ Heart Fail. (2022) 15:e009518. 10.1161/circheartfailure.122.009518 36098058 PMC9588574

[11] Xu R Cui S Chen L Chen XC Ma LL Yang HN Circulating miRNA-1-3p as biomarker of accelerated sarcopenia in patients diagnosed with chronic heart failure. Rev Invest Clin. (2022) 74:276–68. 10.24875/ric.22000151 36328028

[12] Sugita Y Ito K Yoshioka Y Sakai S. Association of complication of type 2 diabetes mellitus with hemodynamics and exercise capacity in patients with heart failure with preserved ejection fraction: a case-control study in individuals aged 65–80 years. Cardiovasc Diabetol. (2023) 22:97. 10.1186/s12933-023-01835-2 37118820 PMC10148403

[13] Fonseca G Dos Santos MR de Souza FR Takayama L Rodrigues Pereira RM Negrão CE Discriminating sarcopenia in overweight/obese male patients with heart failure: the influence of body mass index. ESC Heart Fail. (2020) 7:84–91. 10.1002/ehf2.12545 31877587 PMC7083394

[14] Canteri AL Gusmon LB Zanini AC Nagano FE Rabito EI Petterle RR Sarcopenia in heart failure with reduced ejection fraction. Am J Cardiovasc Dis. (2019) 9:116–26. PMID: PMCID: PMC6971421 31970027 PMC6971421

[15] Dos Santos MR Saitoh M Ebner N Valentova M Konishi M Ishida J Sarcopenia and endothelial function in patients with chronic heart failure: results from the studies investigating comorbidities aggravating heart failure (SICA-HF). J Am Med Dir Assoc. (2017) 18:240–5. 10.1016/j.jamda.2016.09.006 27816483

[16] Saitoh M Dos Santos MR Ebner N Emami A Konishi M Ishida J Nutritional status and its effects on muscle wasting in patients with chronic heart failure: insights from studies investigating co-morbidities aggravating heart failure. Wien Klin Wochenschr. (2016) 128:497–504. 10.1007/s00508-016-1112-8 27853883

[17] Emami A Saitoh M Valentova M Sandek A Evertz R Ebner N Comparison of sarcopenia and cachexia in men with chronic heart failure: results from the studies investigating co-morbidities aggravating heart failure (SICA-HF). Eur J Heart Fail. (2018) 20:1580–7. 10.1002/ejhf.1304 30160804

[18] Bekfani T Pellicori P Morris DA Ebner N Valentova M Steinbeck L Sarcopenia in patients with heart failure with preserved ejection fraction: impact on muscle strength, exercise capacity and quality of life. Int J Cardiol. (2016) 222:41–6. 10.1016/j.ijcard.2016.07.135 27454614

[19] Zhao W Lu M Wang X Guo Y. The role of sarcopenia questionnaires in hospitalized patients with chronic heart failure. Aging Clin Exp Res. (2021) 33:339–44. 10.1007/s40520-020-01561-9 32346826 PMC7914185

[20] Fonseca G Santos MRD Souza FR Costa M Haehling SV Takayama L Sympatho-vagal imbalance is associated with sarcopenia in male patients with heart failure. Arq Bras Cardiol. (2019) 112:739–46. 10.5935/abc.20190061 30970141 PMC6636362

[21] Eschalier R Massoullié G Boirie Y Blanquet M Mulliez A Tartière PL Sarcopenia in patients after an episode of acute decompensated heart failure: an underdiagnosed problem with serious impact. Clin Nutr. (2021) 40:4490–9. 10.1016/j.clnu.2020.12.033 33483182

[22] Lopez PD Nepal P Akinlonu A Nekkalapudi D Kim K Cativo EH Low skeletal muscle mass independently predicts mortality in patients with chronic heart failure after an acute hospitalization. Cardiology. (2019) 142:28–36. 10.1159/000496460 30893691

[23] Katano S Yamano K Yano T Numazawa R Nagaoka R Honma S Prognostic implication of sarcopenia diagnosed by updated Asian working group for sarcopenia criteria in older patients with heart failure: utility and limitation. J Nutr Health Aging. (2025) 29:100434. 10.1016/j.jnha.2024.100434 39642658 PMC12180013

[24] Kılıç R Güzel T Aktan A Güzel H Kaya AF Arslan B Prevalence of sarcopenia in heart failure with mildly reduced ejection fraction and its impact on clinical outcomes. Acta Cardiol. (2024) 79:915–23. 10.1080/00015385.2024.2410604 39377136

[25] Michelini LC O'Leary DS Raven PB Nóbrega AC. Neural control of circulation and exercise: a translational approach disclosing interactions between central command, arterial baroreflex, and muscle metaboreflex. Am J Physiol Heart Circ Physiol. (2015) 309:H381–92. 10.1152/ajpheart.00077.2015 26024683 PMC4631530

[26] Ligthart-Melis GC Luiking YC Kakourou A Cederholm T Maier AB de van der Schueren MAE. Frailty, sarcopenia, and malnutrition frequently (co-)occur in hospitalized older adults: a systematic review and meta-analysis. J Am Med Dir Assoc. (2020) 21:1216–28. 10.1016/j.jamda.2020.03.006 32327302

[27] Zhang Y Zhang J Ni W Yuan X Zhang H Li P Sarcopenia in heart failure: a systematic review and meta-analysis. ESC Heart Fail. (2021) 8:1007–17. 10.1002/ehf2.13255 33576177 PMC8006658

