# Supplementary figures and images for: Comparison of sarcopenia prevalence and prognostic features between HFrEF and HFpEF: a systematic review and meta-analysis
Source: Front Cardiovasc Med. 2025 Nov 17;12:1671305. doi: 10.3389/fcvm.2025.1671305 (PMC12665785; doi:10.3389/fcvm.2025.1671305)

**Figure S1** The flowchart for study screening

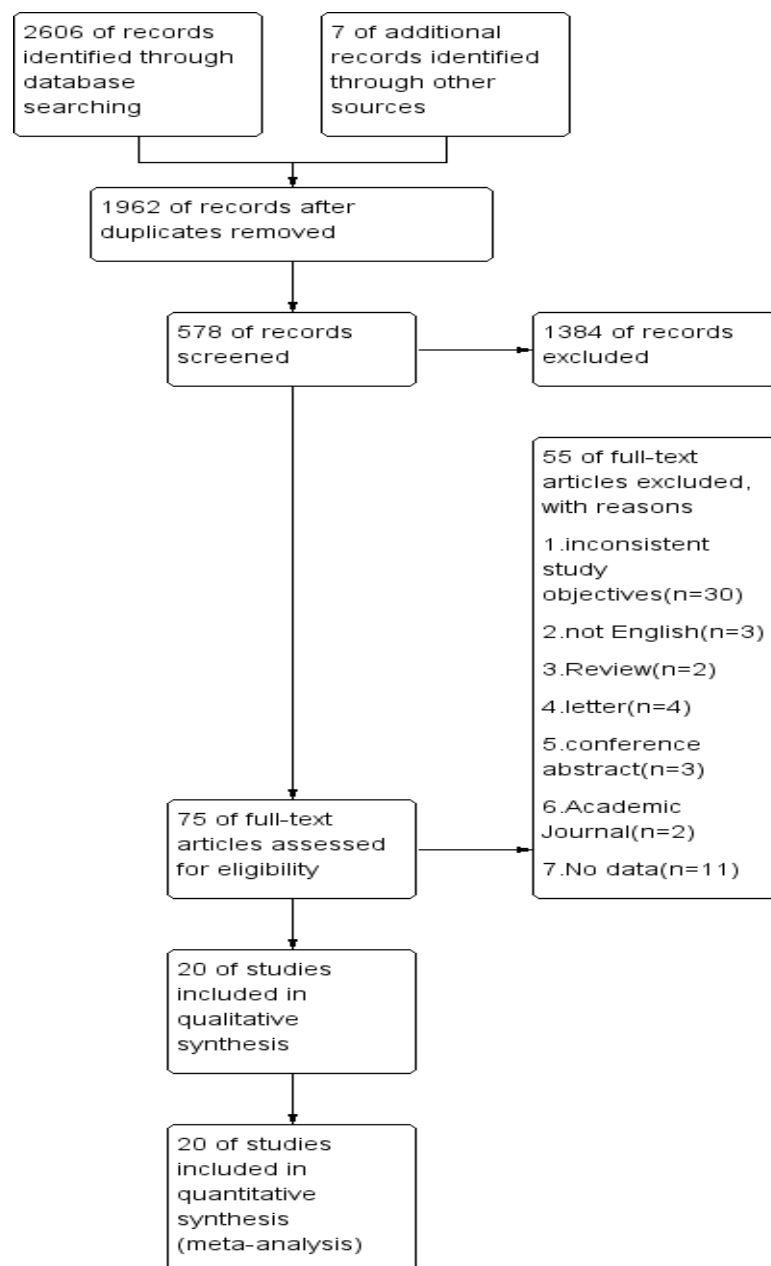

Supplement: Supplementary file 1 [file Table1.pdf]
