# Supplementary material for: Comparison of sarcopenia prevalence and prognostic features between HFrEF and HFpEF: a systematic review and meta-analysis
Source: Front Cardiovasc Med. 2025 Nov 17;12:1671305. doi: 10.3389/fcvm.2025.1671305 (PMC12665785; doi:10.3389/fcvm.2025.1671305)
Supplement: Supplementary file 2 [file Table2.pdf]

Figure S2 Sensitivity analysis of prevalence of sarcopenia in HFrEF and HFpEF

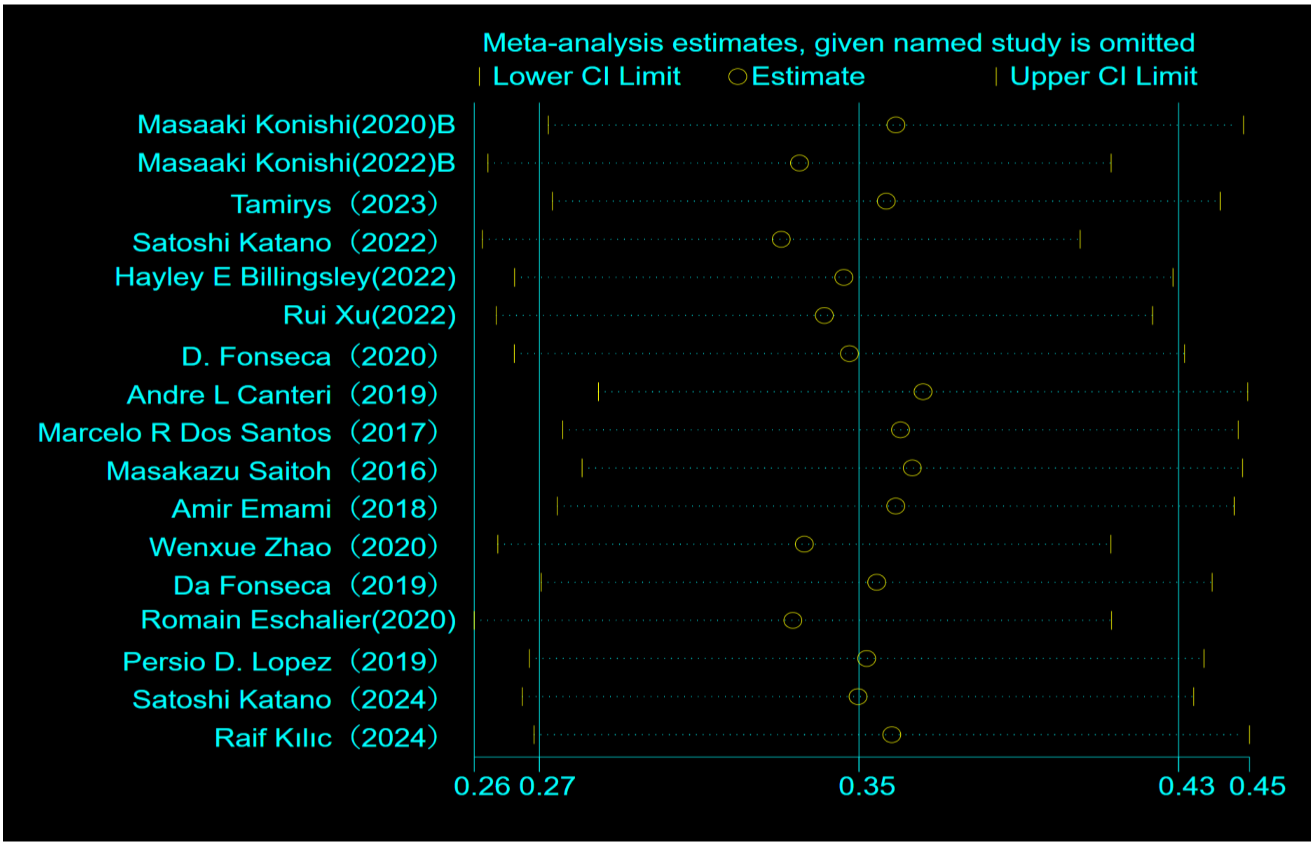

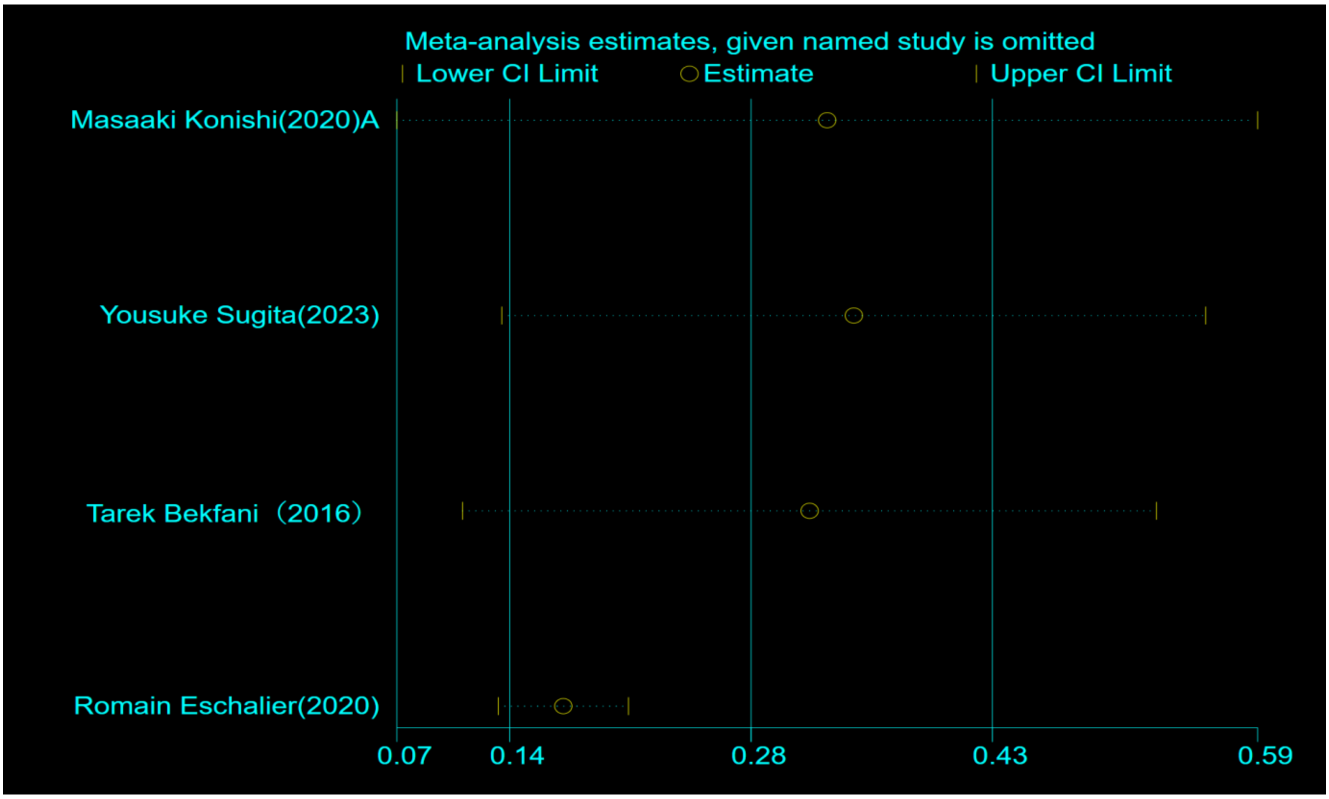

Abbreviations: HFpEF,Heart failure with preserved ejection fraction;HFrEF,Heart failure with reduced ejection fraction.
