# Supplementary material for: Comparison of sarcopenia prevalence and prognostic features between HFrEF and HFpEF: a systematic review and meta-analysis
Source: Front Cardiovasc Med. 2025 Nov 17;12:1671305. doi: 10.3389/fcvm.2025.1671305 (PMC12665785; doi:10.3389/fcvm.2025.1671305)
Supplement: Supplementary file 3 [file Table3.pdf]

Figure S3 Begg's rank correlation test and Egger's regression test

HFrEF:

```
Begg's Test

  adj. Kendall's Score (P-Q) =      36
          Std. Dev. of Score =   24.28
           Number of Studies =      17
                          z  =    1.48
                    Pr > |z| =   0.138
                          z  =    1.44 (continuity corrected)
                    Pr > |z| =   0.149 (continuity corrected)
```

Egger's test

Std_Eff	Coefficient	Std. err.	t	P>|t|	[95% conf.	interval]
slope	.1450748	.1014006	1.43	0.173	-.0710554	.3612049
bias	5.743838	3.355557	1.71	0.108	-1.408363	12.89604

HFpEF:

```
Begg's Test

  adj. Kendall's Score (P-Q) =       4
          Std. Dev. of Score =    2.94
           Number of Studies =       4
                          z  =    1.36
                    Pr > |z| =   0.174
                          z  =    1.02 (continuity corrected)
                    Pr > |z| =   0.308 (continuity corrected)
```

Egger's test

Std_Eff	Coefficient	Std. err.	t	P>|t|	[95% conf.	interval]
slope	.0409371	.1329666	0.31	0.787	-.5311722	.6130464
bias	6.07426	4.757005	1.28	0.330	-14.39348	26.542
